# The soluble form of BMPRIB is a novel therapeutic candidate for treating bone related disorders

**DOI:** 10.1038/srep18849

**Published:** 2016-01-06

**Authors:** Kengo Yamawaki, Yuichiro Kondo, Tsutomu Okada, Takeshi Oshima, Makoto Kakitani, Kazuma Tomizuka

**Affiliations:** 1 Nephrology Research Labs., Nephrology R&D Unit, R&D division, Kyowa Hakko Kirin Co., Ltd., 3-6-6 Asahi-machi, Machida-shi, Tokyo, 194-8533, Japan; 2 Nephrology Research Labs., Nephrology R&D Unit, R&D division, Kyowa Hakko Kirin Co., Ltd., 1188, Shimotogari, Nagaizumi-cho, Sunto-gun, Shizuoka, 411-8731, Japan; 3 Tokyo Research Park, Kyowa Hakko Kirin Co., Ltd., 3-6-6 Asahi-machi, Machida-shi, Tokyo, 194-8533, Japan; 4 R&D division, Oncology R&D Unit, Oncology Research Labs., Kyowa Hakko Kirin Co., Ltd., 1188, Shimotogari, Nagaizumi-cho, Sunto-gun, Shizuoka, 411-8731, Japan; 5 Production division, Bio Process Research and Development Labs., Kyowa Hakko Kirin Co., Ltd., 100-1 Hagiwara-machi, Takasaki-shi, Gunma, 370-0013, Japan; 6 R&D division, Research Functions Unit, Innovative Technology Labs., Kyowa Hakko Kirin Co., Ltd., 3-6-6 Asahi-machi, Machida-shi, Tokyo, 194-8533, Japan

## Abstract

Bone morphogenetic proteins (BMPs) are multi-functional growth factors that belong to the TGF-beta superfamily. Recently, several soluble BMP receptors, such as ActRIIA-Fc, ActRIIB-Fc, and ALK1-Fc, are undergoing clinical trials. Both BMPRIA and BMPRIB are type I BMP receptors, and while BMPRIA-Fc has been reported to have bone-increasing properties, there have been no investigations concerning the biological functions of BMPRIB-Fc. Therefore, comparing the effects of BMPRIA-Fc and BMPRIB-Fc *in vivo* should be helpful in revealing the differences in biological function between BMPRIA and BMPRIB, and would also aid in the evaluation of BMPRIB-Fc as a therapeutic agent. Here, we produced Tg chimeras in which BMPRIA-Fc and BMPRIB-Fc proteins circulated at high concentrations (36.8–121.4 μg/mL). Both Tg chimeras showed a significant increase of bone volume and strength. Using histological analysis, adenoma of the glandular stomach was observed only in BMPRIA-Fc chimeras suggesting the tumorigenic activity of this protein. Administration of recombinant BMPRIB-Fc protein to normal mice also increased bone volumes. Finally, treatment with BMPRIB-Fc decreased the area of osteolytic regions in a mouse model of breast cancer metastasis. In conclusion, our data suggest that BMPRIB-Fc can be used for the treatment of bone-related disorders with a lower risk than BMPRIA-Fc.

Bone morphogenetic proteins (BMPs) and growth differentiation factors (GDFs), secreted proteins belonging to the TGF-beta superfamily, are pleiotropic factors that play a variety of roles in the regulation of embryonic development and postnatal homeostasis of various organs and tissues by controlling cellular differentiation, proliferation, and apoptosis. In mammals, there are more than 20 BMP/GDF ligands, 7 type I receptors (activin receptor-like kinase [ALK] 1–7), and 5 type II receptors (ActRIIA, ActRIIB, BMPRII, TGFβRII, and MISIIR). The BMP/GDF ligands transduce signals through a complex of type I and II serine/threonine kinase receptors via Smad-dependent and -independent cascades that eventually evoke the transcriptional activation of downstream target genes^1^,^2^,^3^.

To elucidate the physiological functions of BMP/GDF ligands and their receptors *in vivo*, phenotypic analyses using knockout (KO) mice have been widely employed^4^,^5^. In addition, ligand trapping with soluble receptors is considered an alternative “loss of function” approach to study the effects of blocking the signaling mediated by BMP/GDF ligand-receptor interactions^3^. For example, the administration of ActRIIA-Fc and ActRIIB-Fc to mice results in increased bone volume (BV), number of red blood cells (RBCs), and muscle hypertrophy through activin-A or GDF8 trapping^6^,^7^,^8^. ALK1-Fc is used as an anti-angiogenic agent in tumor suppression^9^ and the administration of BMPRIA-Fc increases BV through BMP2/4 trapping^10^. Ligand trapping strategies using Fc-fusion proteins are widely used for therapeutic purposes, for example etanercept (a soluble form of the tumor necrosis factor receptor), which is already available for human use, and ActRIIA-Fc, ActRIIB-Fc, and ALK1-Fc are now also undergoing clinical trials for the treatment of anemia, bone-related diseases, and cancer^11^,^12^,^13^,^14^.

Both BMPRIA (ALK3) and BMPRIB (ALK6) belong to the type I receptor family, and play several roles in the biological effects of R-Smad (Smad1, 2, 3, 5, and 8) signaling^1^,^2^. These 2 receptors bind several BMP/GDF ligands in common^15^,^16^ and have overlapping functions, such as in chondrogenesis^17^ and ovary formation^18^. On the other hand, some ligands such as GDF9/BMP15 preferentially bind to BMPRIB^19^, and the 2 receptors play different roles in the nervous system^20^. The functional differences between BMPRIA and BMPRIB are still unclear.

Previous studies in bone formation using such as KO, conditional-KO (cKO) and transgenic (Tg) mice suggest that inhibiting signal transduction via BMPRIA acts to “increase” bone formation, whereas inhibiting BMPRIB-signal acts to “decrease” bone formation^21^,^22^,^23^,^24^,^25^,^26^.

Comparing the function of BMPRIA-Fc and BMPRIB-Fc *in vivo* should be helpful in elucidating any differences in biological function between BMPRIA and BMPRIB. In this report, we generated BMPRIA-Fc and BMPRIB-Fc Tg chimeric mice using our own Tg system, as previously published. Using this method, we were the first group to successfully identify the potent and specific proliferative effects of R-spondin1 on intestinal crypt cells. In addition, we discovered some novel phenotypes caused by BMP type II receptor-Fc proteins (ActRIIA-Fc, ActRIIB-Fc, and BMPRII-Fc), such as an increased number of RBCs, extramedullary hematopoiesis in the spleen, increased bone formation, and decreased viability in adults^3^,^27^,^28^. The average serum concentrations of BMPRIA-Fc and BMPRIB-Fc in the Tg chimeras were 53.1 μg/mL and 88.1 μg/mL respectively, which are much higher than those of endogenous BMP/GDF ligands. Both Tg chimeras showed significant increases in BV and bone strength without increased incidence of death at 16 weeks of age. Administration of the recombinant BMPRIB-Fc protein to normal mice increased BV. Treatment with BMPRIB-Fc also decreased the area of osteolytic regions in a mouse model of breast cancer metastasis. Unexpectedly, histological analysis revealed that adenoma of the glandular stomach formed in BMPRIA-Fc chimeras at 16 weeks of age, but not in BMPRIB-Fc chimeras. Our data suggest that BMPRIB-Fc can be used for the treatment of bone-related disorders with a lower risk than BMPRIA-Fc.

## Results and Discussion

### Generation of Tg chimeras

**Figure 1:**
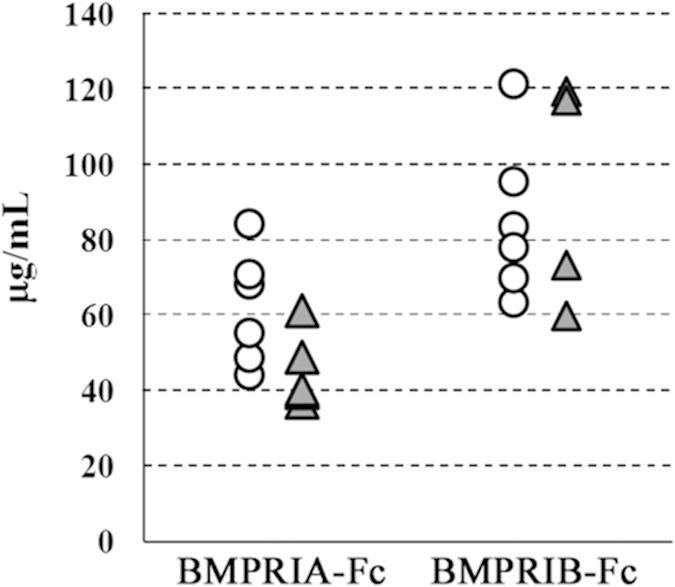
The transgene expression level of the Tg chimeras. Serum concentrations of each soluble protein (BMPRIA-Fc and BMPRIB-Fc) in 16-week-old transgenic chimeras were measured using ELISA (see Methods). White circles represent females, and gray triangles represent males. In the control chimeras, the Fc protein was not detectable.

First, we produced BMPRIA-Fc and BMPRIB-Fc Tg chimeras using our original system. In the Tg chimeras, BMPRIA-Fc and BMPRIB-Fc proteins were found to be circulating at very high levels in 16 week-old mice, as measured by ELISA (averages of 53.1 μg/mL (=0.66 μM) and 88.1 μg/mL (=1.1 μM), respectively; Fig. 1). In a previous study, transgenic mice expressing TGFbRII-Fc under the regulation of the mammary-selective MMTV-LTR promoter/enhancer^29^, for example, the average serum concentration of the protein was 400 ng/mL. Compared to such a report, our Tg chimeras can be seen to be achieving very high transgene expression levels.

### Bone morphometry and bone strength test of the Tg chimeras

**Figure 2:**
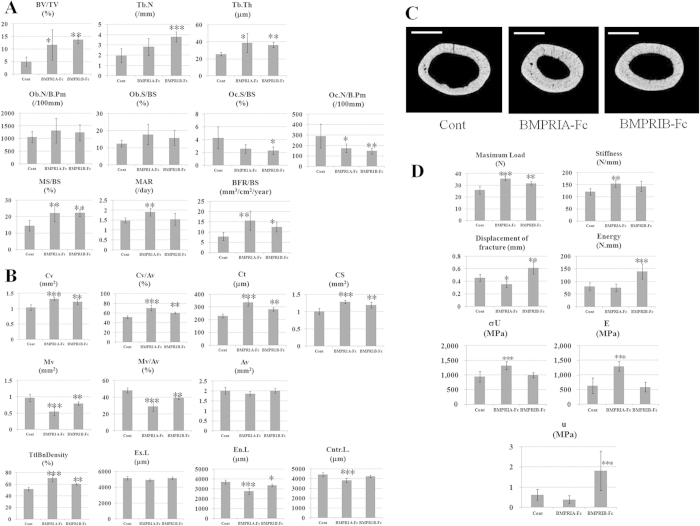
Bone morphometry and bone strength test of the Tg chimeras. Bone parameters and bone strength of each female Tg chimera (N = 6) at 16 weeks of age. (**A**) Trabecular bone parameters, (**B**,**C**) Cortical bone parameters and the 2D micro CT images, and (**D**) Bone strength. Femur samples were used for measurement of bone strength and cortical bone parameters and tibia samples were used for trabecular bone parameters. BV/TV: bone volume/tissue volume, Ob.N/B.Pm: osteoblast number/bone perimete, Ob.S/BS: osteoblast surface/bone surface, MAR: mineral apposition rate, MS/BS: mineralization surface/bone surface, BFR/BS: bone formation rate/bone surface, Oc.N/B.Pm: osteoclast number/bone perimeter, Oc.S/BS: osteoclast surface/bone surface, Tb.Th: trabecular thickness, Tb.N: trabecular number, Cv: Cortical bone volume, Mv: Medullary volume, Av: All bone volume, Cv/Av: Cortical bone ratio, Mv/Av: medullary rtio, Ct: Cortical bone thickness, CS: Cortical bone section area, TtlBnDensity:total bone density, Ex.L: External length, En.L: Endosteal length, Cntr.L.: Center line length, σ_U_: ultimate stress, E: Young’s modulus, u: modulus of toughness. All results are presented as the mean ± SD. *P < 0.05 vs control; **P < 0.01 vs control; ***P < 0.001 vs control (Dunnett-test). Scale bars indicate 0.5 mm.

Figure 2 shows the trabecular bone parameters of tibiae and the cortical bone parameters, 2D microCT images bone strength of femora obtained from each female Tg chimera at 16 weeks of age. In all Tg chimeras, an increase of bone strength, bone volume/tissue volume (BV/TV), trabecular thickness (Tb.Th), trabecular number (Tb.N), mineralization surface/bone surface (MS/BS), and bone formation rate/bone surface (BFR/BS) of the trabecular bone were observed (Fig. 2A). The increase of BV/TV observed in our BMPRIA-Fc Tg chimera corresponds to a previous report^10^, suggesting that the soluble protein forms in the Tg chimeras were effective, confirming the validity of our system.

We found for the first time that BMPRIB-Fc has a bone-increasing effect *in vivo*. Bone morphometry analysis demonstrated that osteoclastic functions (osteoclast number/bone perimeter as the bone resorption parameter (Oc.N/B.Pm), osteoclast surface/bone surface (Oc.S/BS)) were suppressed, while there were no obvious changes in osteoblastic functions (osteoblast number/bone perimeter as the bone formation parameter (Ob.N/B.Pm), osteoblast surface/bone surface (Ob.S/BS)). These results suggest that unbalanced bone remodeling, especially decreased osteoclastic bone resorption, may lead to an increase in BV. The cortical bone parameters such as cortical bone volume (Cv), cortical bone ratio (Cv/Av), cortical bone thickness (Ct), cortical bone section area (CS), total bone density (TtlBnDensity) were increased while medullary volume (Mv), medullary ratio (Mv/Av), endosteal length (En.L) were decreased (Fig. 2B,C). In addition, the three-point bending test showed increased maximun load and stiffness (Fig. 2D). Those data suggest that bone strength also increased. In the BMPRIB-Fc chimeras, stiffness did not increase significantly while both displacement of fracture and energy increased, suggesting the toughness, rather than stiffness, was increased. Further, such difference about bone quality between BMPRIA-Fc and BMPRIB-Fc is supposed by other mechanical parameters such as ultimate stress (σ_U_), Young’s modulus (E) and modulus of toughness (u)^30^. Previous studies regarding BMPRIB, including KO, Tg, and *in vitro* methods, all suggest that signal transduction via BMPRIB causes bone formation^23^,^24^,^26^, therefore it was surprising to observe that suppression of the signal by ligand trapping with BMPRIB-Fc leads to an increase in BV and bone strength. The relationship of sclerostin or Wnt ligands to the bone increasing phenotype remains to be elucidated and it’s necessary future work.

### Histological analysis

**Figure 3:**
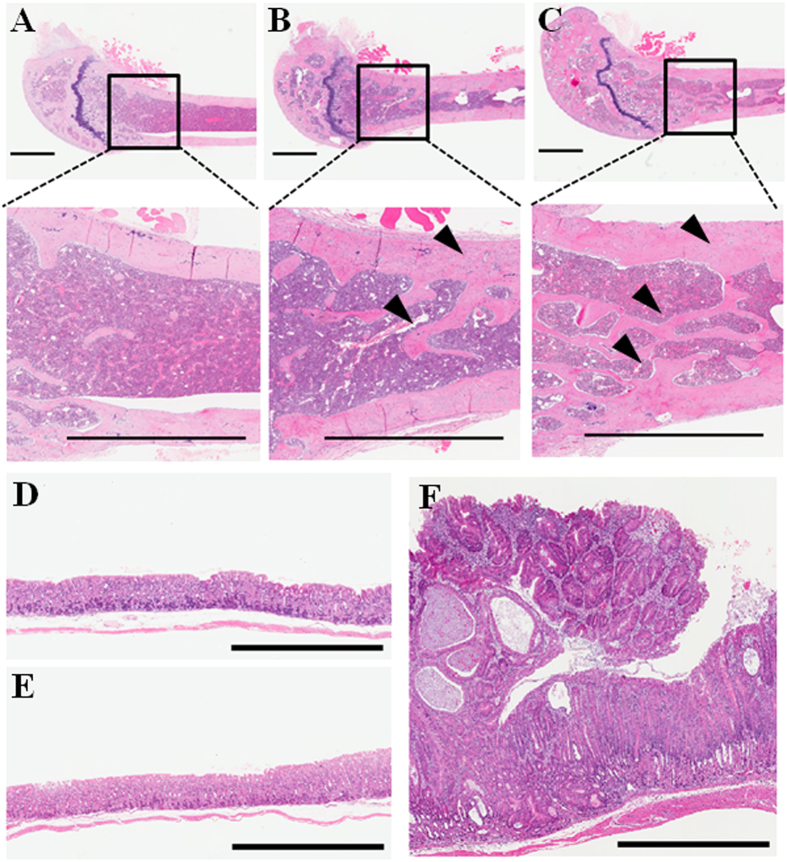
Histological analysis of the Tg chimeras. Histological images of the femur (**A**–**C**) and stomach (**D**–**F**) of each Tg chimera at 16 weeks of age. (**A**,**D**) control; (**B**,**F**) BMPRIA-Fc; and (**C**,**E**) BMPRIB-Fc. The arrow heads represent regions of increased trabecular bone and cortical bone thickness in BMPRIA-Fc and BMPRIB-Fc Tg chimeras (**B**,**C**). Adenoma of the glandular stomach was observed only in the BMPRIA-Fc Tg chimera (**F**). All scale bars indicate 1 mm.

To elucidate the biological effect of BMPRIB-Fc on tissues other than bone, histological analysis with hematoxylin-eosin staining was performed (Fig. 3). An increase of trabecular bone was observed in both BMPRIA-Fc and BMPRIB-Fc mice, which corresponds to the data of bone morphometry. Though there were no obvious findings for BMPRIB-Fc Tg chimeras in other tissues (brain, heart, lung, thymus, mesenteric lymph nodes, ovary, testis, pancreas, liver, adrenal gland, kidney, stomach, and spleen), adenoma of the glandular stomach was observed in BMPRIA-Fc chimeric mice. This was surprising because, such a phenotype was not reported in the case of BMPRIA-Fc administration in mice^10^. Though further study will be required to elucidate the mechanism of this phenotype, some previous work suggests that BMPRIA signaling is related to tumorigenesis. For example, Howe *et al.* reported that genetic mutation of BMPRIA, not BMPRIB, was found in patients with juvenile polyposis^31^, while Voorneveld *et al.* showed that reduced expression of BMPRIA in pancreatic cancer is associated with a poor prognosis^32^, and Bleuming *et al.* demonstrated that Mx1-Cre-Bmpr1a^−/−^ mice develop intestinal polyposis and neoplasia at gastric junctional zones^33^,^34^. The binding affinities for each ligand differed in some cases between BMPRIA-Fc and BMPRIB-Fc according to SPR data (Table 1), and future work should determine whether such differences result in tumorigenesis. Our approach, which enables systemic and long-term circulation of proteins in the mouse body, would be useful in toxicological assessment of recombinant protein therapeutics. From such a point of view, our data suggest that BMPRIB-Fc exhibits lower toxicity than BMPRIA-Fc.

The results of our ligand-trap strategy may be affected by different circumstances (e.g. mouse strains). Therefore it should be noted that the bone increase phenotype and toxic phenotype (i.e. adenoma of the glandular stomach) is possibly specific for this study. However, our Tg chimera shows chimerism-independent, B-cell-specific transgene expression. Actually, though the chimerism of the Tg mice in this study is ranged 5–80%, bone increase phenotype were consistently observed in all of the BMPRIA-Fc and BMPRIB-Fc Tg chimeras and the adenoma of the glandular stomach was observed in all BMPRIA-Fc chimeric mice, not in BMPRIB-Fc chimeras. This may suggest the phenotypes seen in this study is independent from mouse strain, but further study will be necessary using germline transmittants with cleaner genetic background.

### Treatment of normal mice with recombinant BMPRIB-Fc

**Figure 4:**
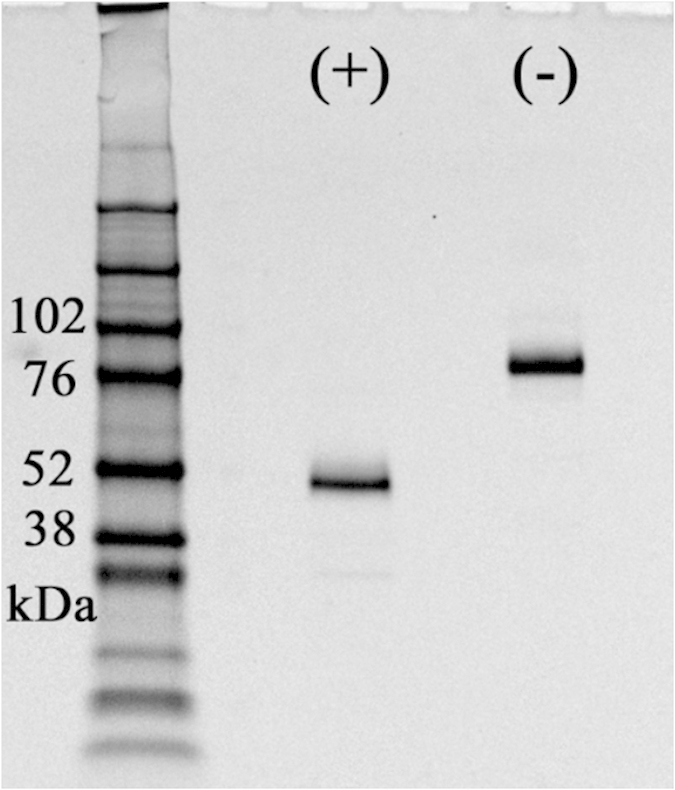
CBB-stained SDS-PAGE gel image of recombinant BMPRIB-Fc. (+) lane represents reducing conditions and (–) lane represents non-reducing conditions. 1 μg/lane of BMPRIB-Fc protein was applied.

To confirm the results with the BMPRIB-Fc Tg chimera, we prepared recombinant BMPRIB-Fc protein and performed *in vivo* experiments. First, we produced and purified the BMPRIB-Fc recombinant protein using 293F cells and Protein A columns (see Methods). Then, using SDS-PAGE, a clear single band was observed at 45–50 kDa under reducing conditions, and at 90–100 kDa under non-reducing conditions (Fig. 4), suggesting a disulfide-linked homodimer. Subsequently, we evaluated the ligand affinity of the recombinant protein using SPR and confirmed that our proteins bind to several known BMP/GDF ligands, such as BMP2/4 (Table 1).

**Figure 5:**
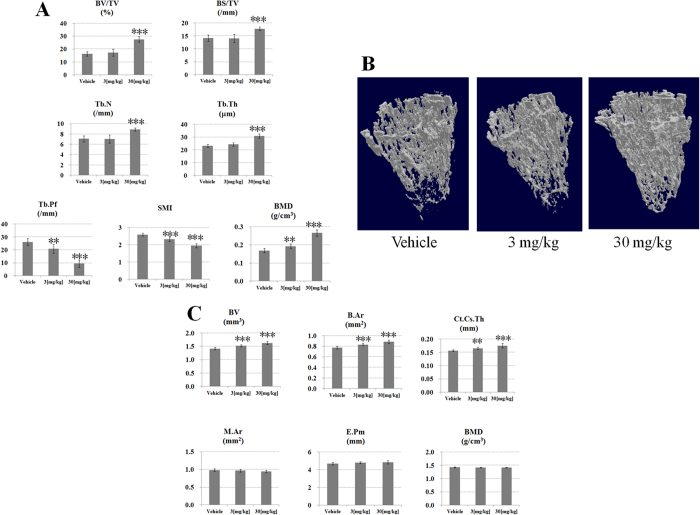
Bone parameters and 3D microCT images of BMPRIB-Fc-administered C57BL/6 mice. (**A**) Trabecular bone parameters (**B**) 3D micro CT images of trabecular bone (**C**) Cortical bone paremeters. BV/TV (bone volume/tissue volume), BS/TV (bone surface/tissue volume), BMD (bone mineral density), Tb.Pf (Trabecular pattern factor), SMI (structure model index), Tb.Th (Trabecular thickness), Tb.N (Trabecular number), B.Ar (Mean total crossectional bone area), Ct.Cs.Th (Cortical crossectional thickness), M.Ar (Medullary area), E.Pm (Endosteal perimeter). All results are presented as the mean ± SD. *P < 0.05 vs vehicle; **P < 0.01 vs vehicle; ***P < 0.001 vs vehicle (williams-test).

Next, we performed an *in vivo* administration experiment on normal mice. The recombinant protein was administered to 8 week-old male C57BL/6 mice at a dose of 3 or 30 mg/kg (q10d3) and as a result, BMD of trabecular bone and BV, mean total cross-sectional bone area (B.Ar) and cross-sectional thickness (Ct.Cs.Th) of cortical bone was increased in a concentration-dependent manner (Fig. 5). At a dose of 30 mg/kg, BV/TV, BS/TV, Tb.Th, and Tb.N of trabecular bone also increased significantly. Trabecular pattern factor (Tb.Pf) is an index for trabecular bone connectivity, and the structure model index (SMI) indicates the relative prevalence of rods and plates in the trabecular bone. These parameters decreased in a concentration-dependent manner, suggesting that the structural integrity and mechanical strength of the trabecular bone was improved and the mice have greater bone connectivity and healthier plate-like bone structures by administration of recombinant BMPRIB-Fc. In previous reports, blocking BMP2/4 signaling by administering BMPRIA-Fc caused an early increase in osteoblast number with short-term treatment, and a late decrease in osteoclast number with long-term treatment^10^. BMPRIB-Fc has a strong binding affinity to BMP2/4, as does BMPRIA-Fc (Table 1). The Tg chimera, which corresponds to long-term treatment with BMPRIB-Fc, showed a decrease in osteoclast number but not increase in osteoblast number, suggesting that the bone-increasing mechanism of BMPRIB-Fc is the same as that of BMPRIA-Fc. Further study using recombinant BMPRIB-Fc protein will be required.

### Administration of BMPRIB-Fc to breast cancer bone metastasis-model mice

**Figure 6:**
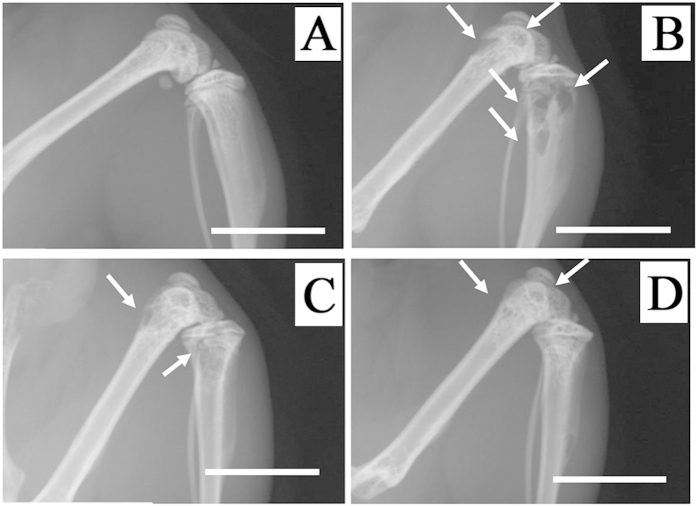
X-ray photographic images of femora in a mouse model of breast cancer bone metastasis. (**A**) Normal mouse, (**B**) breast cancer metastasis model mouse treated with Vehicle, (**C**) mouse treated with 3 mg/kg of BMPRIB-Fc, (**D**) mouse treated with 30 mg/kg of BMPRIB-Fc. White arrows represent osteolytic regions. All scale bars indicate 5 mm.

**Figure 7:**
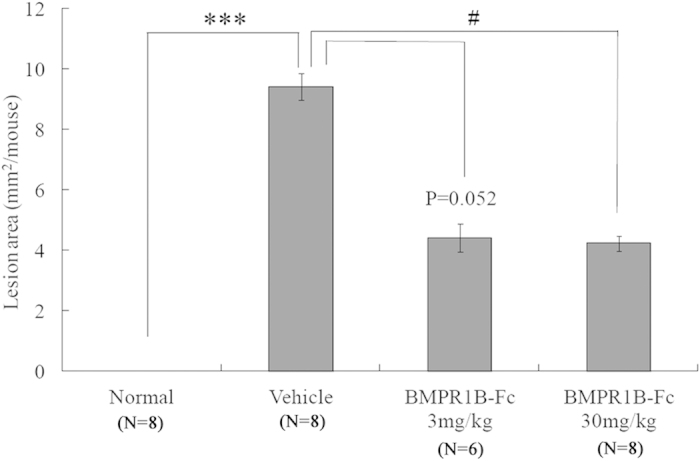
Quantification of osteolytic lesion areas in a mouse model of breast cancer bone metastasis. The total lesion areas of left/right femora, tibiae and humeri are depicted. All results are presented as the mean ± SE. ***P < 0.001 vs normal (Student’s *t*-test); ^#^P < 0.05 vs vehicle (Steel’s test).

Finally, we investigated whether BMPRIB-Fc can be used as a potential therapeutic agent to treat bone diseases by using a mouse model of breast cancer bone metastasis. Seventy-five percent of breast cancer patients have bone metastasis, of which 80% have a reduced quality of life caused by bone pain and skeletal complications, therefore there is a great therapeutic need^35^. From X-ray photographic analysis (Fig. 6), bone destruction was observed in MDB-MB-231 cell-inoculated mice. However, the region of bone destruction was smaller in mice administered with BMPRIB-Fc than in those treated with PBS. Quantitative analysis revealed that BMPRIB-Fc decreased the region of bone destruction in a concentration-dependent manner (Fig. 7), suggesting that BMPRIB-Fc demonstrates therapeutic properties. It is not clear whether BMPRIB-Fc can prevent bone metastasis of cancer cells. Chantry *et al.* reported that ActRIIA-Fc could prevent bone metastasis in mice carrying MDA-MB-231 cells, and suggested that such effects are mediated indirectly via changes in the bone microenvironment^6^. To discuss this point, we checked the expression of BMPRIB of MDA-MB-231 cells and also tested whether BMPRIB-Fc could affect the proliferation of MDA-MB-231 cells. As a result, MDA-MB-231 cells does not express BMPRIB and BMPRIB-Fc did not affect the proliferation of them (data not shown). This suggest that the decrease of bone destruction by BMPRIB-Fc may be due to its bone increasing effect rather than the direct effect to the MDA-MB-231 cells. However, Other studies have suggested that BMP2 and BMP4, high-affinity ligands of BMPRIB-Fc, can promote the invasion, migration, and microcalcification of breast cancer cells^36^,^37^,^38^, and Helms *et al.* reported that strong expression of BMPRIB was associated with high tumor grade and a poor prognosis in estrogen receptor-positive breast cancers^39^. Further study will be necessary to conclude that BMPRIB-Fc did not affect MDA-MB-231 directly.

In conclusion, we found that BMPRIB-Fc shows a bone-increasing effect without marked toxicity and reduces the area of bone destruction caused by breast cancer metastasis. Thus, BMPRIB-Fc may constitute a therapeutic agent for the treatment of bone-related diseases.

## Methods

### Generation of Tg chimeras

All animal procedures were performed in accordance with the protocols approved by the Institutional Animal Care and Use Committee of Kyowa Hakko Kirin Co., Ltd. (Approval Number: A-95). Chimeric mice expressing BMPRIA-Fc and BMPRIB-Fc fusion proteins were generated as described in ref. 3. Briefly, we used the immunoglobulin kappa (Igk) promoter and inserted the transgene units into the genomic site adjacent to the endogenous Igk locus of murine embryonic stem (ES) cells using homologous recombination. The resultant ES cell clones were injected into embryos derived from a B-cell-deficient host strain, thus resulting in chimerism-independent, B-cell-specific transgene expression. In this study, the “control” chimeras were produced using ES cells in which the expression unit was not introduced. The cDNA fragments of the extracellular domains of BMPRIA (NM_009758.4, 70–456 bp) and BMPRIB (NM_007560.3, 4–378 bp) were obtained from FANTOM cDNA Clones (RIKEN).

### Measurement of the serum concentrations of BMPRIA-Fc and BMPRIB-Fc using ELISA

Serum was obtained from 16-week-old Tg chimeric mice, and protein levels produced by the transgenes were measured using ELISA. Anti-hIgG (gamma-chain specific; SIGMA) was immobilized on a plate and detected with peroxidase-conjugated anti-hIgG (Fc-specific; SIGMA). hFc (IgG1) was used as a standard. For normalization, the measured value was multiplied by the ratio of the molecular weights of the soluble-form proteins divided by that of hFc (IgG1). All animal procedures were performed in accordance with the protocols approved by the Institutional Animal Care and Use Committee of Kyowa Hakko Kirin Co., Ltd. (Approval Number: A-95).

### Histological analysis

Chimeric animals were sacrificed under ether anesthesia for histological assessment of organs (brain, heart, lung, thymus, mesenteric lymph nodes, femur, ovary, testis, pancreas, liver, adrenal gland, kidney, stomach, and spleen), and hematoxylin-eosin staining was carried out. All animal procedures were performed in accordance with the protocols approved by the Institutional Animal Care and Use Committee of Kyowa Hakko Kirin Co., Ltd. (Approval Number: A-95).

### Bone morphometry

In order to obtain data regarding mineral apposition rate (MAR), mineralization surface (MS), and BFR, calcein (Dojindo), a calcium chelator, was dissolved in 2% sodium bicarbonate (Kanto Chemical Co., Inc.), and the prepared solution was administered subcutaneously at a dose of 16 mg/kg prior to necropsy. Calcein was administered 6 days or 1 day before necropsy to 16 week-old female Tg chimeric mice.

Tibiae were sampled from Tg chimeric mice at necropsy, samples of undemineralized tibial sections were prepared, and the samples were then subjected to toluidine blue (TB), alkaline phosphatase (AP), and tartrate-resistant acid phosphatase (TRAP) staining. In order to prepare section samples, the tibiae were embedded in glycol methacrylate (GMA) resin in advance. The metaphyseal secondary cancellous bones (0.3–1.5 mm from growth plates) of the obtained samples of undemineralized sections were subjected to the following measurements using Osteoplan II (Carl Zeiss): bone volume/tissue volume as the bone structure parameter (BV/TV), osteoblast number/bone perimeter as the bone formation parameter (Ob.N/B.Pm), osteoblast surface/bone surface (Ob.S/BS), MAR, mineralization surface/bone surface (MS/BS), bone formation rate/bone surface (BFR/BS), osteoclast number/bone perimeter as the bone resorption parameter (Oc.N/B.Pm), osteoclast surface/bone surface (Oc.S/BS), trabecular thickness (Tb.Th), and trabecular number (Tb.N). BX51 (Olymbus) was used as the microscope and the object lens magnification was X2.

For measurement of the cortical bone parameter, a site 50% away from the proximal end of the femur was subjected to 2D micro-CT photographing, and the cross-sectional area was subjected to the following measurement using Scan Xmate-L090 (Comscantecno) and 3D-BON (Ratoc system engineering): cortical bone volume (Cv), medullary volume (Mv), all bone volume (Av), cortical bone ratio (Cv/Av), medullary ratio (Mv/Av), cortical bone thickness (Ct), cortical bone section area (CS), total bone density (TtlBnDensity), external length (Ex.L), endosteal length (En.L), and center line length (Cntr.L).

All animal procedures were performed in accordance with the protocols approved by the Institutional Animal Care and Use Committee of Kyowa Hakko Kirin Co., Ltd. (Approval Number: A-95).

### Measurement of Bone Strength

Femur samples were obtained at necropsy and wrapped in gauze soaked in saline, then immediately frozen until they were subjected to a three-point bending test using MZ-500S (Marto). Before measurement, samples were thawed by placing room temperature over 2 hours. When conducting a test, the span of the support points was set to 6 mm, and a load was applied at the midpoint of the span at head-speed of 10 mm/min to measure the maximum load (N), Stiffness (N/mm) displacement of fracture (mm), energy absorbed to failure (N.mm), ultimate stress (σU), Young’s modulus (E) and modulus of toughness (u). All animal procedures were performed in accordance with the protocols approved by the Institutional Animal Care and Use Committee of Kyowa Hakko Kirin Co., Ltd. (Approval Number: A-95).

### Recombinant BMPRIB-Fc production

The expression vector was constructed by introducing a BMPRIB-Fc PCR fragment (amplified using the following primers: Fw, CGGGATCCACCATGGAGACAGACAC; Rv, ATAGTTTAGCGGCCGCTCATTTACCCGGAGACAGG) into the BamHI-NotI site of the pLN1 vector^40^. The expression vector was transfected into 293F cells (Invitrogen) and cultured at 37 °C in 5% CO_2_. Three days later, the culture supernatant was collected and filtrated, and BMPRIB-Fc protein was purified using Protein A columns (GE Healthcare). The solvent was exchanged to PBS using NAP-25 columns (GE Healthcare) and sterilized with a 0.22 μm filter. Analysis of purified proteins was carried out using SDS-PAGE and Coomassie Brilliant Blue staining, under reducing and non-reducing conditions.

### Ligand binding affinity of BMPRIB-Fc

Receptor-ligand binding affinities were determined by surface plasmon resonance (SPR), as previously described^10^ with slight modifications: we used HBS-EP (+) buffer as running buffer, an anti-human Fc IgG capture kit for capturing BMPRIA-Fc and BMPRIB-Fc, and the ligands were injected at 25 °C at a flow rate of 30 μL/min. We used a single-cycle kinetics assay to determine the binding affinities of BMPRIA-Fc and BMPRIB-Fc, and association and dissociation phase data were analyzed by kinetics using a 1:1 binding model in Biacore T100 Evaluation Software (GE Healthcare). Kinetic constants were calculated as the average of 2 or 3 independent analyses of receptor-ligand interactions. BMPRIA-Fc, and human BMP2, BMP4, BMP6, BMP7, BMP9, BMP10, BMP15, Activin A, and GDF11, and mouse GDF-5, GDF-6, and GDF-7 were purchased from R&D Systems. Human GDF9 was purchased from Biovision.

### Analysis of the *in vivo* effect of recombinant BMPRIB-Fc

All animal procedures were performed in accordance with the protocols approved by the Institutional Animal Care and Use Committee of Kyowa Hakko Kirin Co., Ltd. (Approval Number: A-123). Recombinant BMPRIB-Fc (3 mg/kg or 30 mg/kg) or PBS was administered intravenously to male 8-week-old C57BL/6 mice on Day 0, Day 10 and Day 20. Mice were sacrificed on Day 30 and femoral bones were collected. The bones were fixed with 70% ethanol, then bone parameters such as BV/TV (bone volume/tissue volume), BS/TV (bone surface/tissue volume), BMD (bone mineral density), Tb.Pf (Trabecular pattern factor), SMI (structure model index), Tb.Th (Trabecular thickness), Tb.N (Trabecular number) of trabecular bone and BV, B.Ar (Mean total crossectional bone area), Ct.Cs.Th (Cortical crossectional thickness), M.Ar (Medullary area), E.Pm (Endosteal perimeter) and BMD of cortical bone were measured using a SkyScan 1174 scanner (SkyScan). Data analysis was performed using CTAn and CTVol (SkyScan).

### Breast cancer bone metastasis model

All animal procedures were performed in accordance with the protocols approved by the Institutional Animal Care and Use Committee of Kyowa Hakko Kirin Co., Ltd. (Approval Number: 10–042). As described in a previous report^35^, breast cancer bone metastasis-model mice were produced by inoculating MDA-MB-231 cells (5 × 10^5^ cells/0.1 mL) into the left cardiac ventricle of female 6 week-old CB17/Icr-Prkdc<scid>/CrlCrlJ mice. Multiple administration of recombinant BMPRIB-Fc was started after one day (Day 1) from the date of the inoculation. BMPRIB-Fc (3 mg/kg or 30 mg/kg) or PBS was repeatedly administered intravenously on Day 1, Day 11, Day 21, and Day 31. Mice were sacrificed on Day 35 and femora, tibiae, and humeri were analyzed by X-ray photography.

### Statistical Analysis

Data of bone parameters were analyzed by one-way ANOVA with Dunnett’s post hoc test (Fig. 2), by one-way ANOVA with William’s post hoc test (Fig. 5). Data of osteolytic lesion area of the breast cancer bone metastasis model experiment (Fig. 7) were analyzed by Student’s t-test (Normal vs Vehicle) and by Kruskal wallis with Steel’s post hoc test (Vehicle vs BMPRIB-Fc administration group). All statistical analyses were performed using SAS 9.2 (SAS Institute Inc).

## Additional Information

**How to cite this article**: Yamawaki, K. *et al.* The soluble form of BMPRIB is a novel therapeutic candidate for treating bone related disorders. *Sci. Rep.* **6**, 18849; doi: 10.1038/srep18849 (2016).

## Figures and Tables

**Table 1:** Binding affinity (K_D_, [M]) of each BMP ligands to BMPRIA-Fc and BMPRIB-Fc.

	BMPRIA-Fc	BMPRIB-Fc
BMP2	3.75 ± 0.82 × 10^−12^	2.48 ± 0.06 × 10^−12^
BMP4	5.64 ± 0.26 × 10^−11^	2.21 ± 0.01 × 10^−10^
BMP6	7.92 ± 1.07 × 10^−11^	0.35 ± 0.29 × 10^−12^
BMP7	1.95 ± 0.05 × 10^−9^	2.68 ± 0.12 × 10^−10^
GDF5	1.63 ± 0.03 × 10^−9^	1.29 ± 0.08 × 10^−10^
GDF6	4.80 ± 0.01 × 10^−10^	1.59 ± 0.05 × 10^−10^
GDF7	4.32 ± 0.06 × 10^−10^	2.09 ± 0.05 × 10^−10^
BMP10	No binding	9.45 ± 0.54 × 10^−12^
BMP9	No binding	No binding
BMP15	No binding	No binding
GDF9	No binding	No binding
GDF11	No binding	No binding
Activin-A	No binding	No binding
